# RNA-Seq *de novo* assembly and differential transcriptome analysis of the nematode *Ascaridia galli* in relation to *in vivo* exposure to flubendazole

**DOI:** 10.1371/journal.pone.0185182

**Published:** 2017-11-03

**Authors:** Mihaela M. Martis, Behdad Tarbiat, Eva Tydén, Désirée S. Jansson, Johan Höglund

**Affiliations:** 1 National Bioinformatics Infrastructure Sweden (NBIS), Department of Clinical and Experimental Medicine, Division of Cell Biology, Linköping University, Linköping, Sweden; 2 Swedish University of Agricultural Sciences (SLU), Department of Biomedical Sciences and Veterinary Public Health, Section for Parasitology, Uppsala, Sweden; 3 National Veterinary Institute (SVA), Department of Animal Health and Antimicrobial Strategies, Uppsala, Sweden; New England Biolabs Inc, UNITED STATES

## Abstract

The nematode *Ascaridia galli* (order Ascaridida) is an economically important intestinal parasite responsible for increased food consumption, reduced performance and elevated mortality in commercial poultry production. This roundworm is an emerging problem in several European countries on farms with laying hens, as a consequence of the recent European Union (EU) ban on conventional battery cages. As infection is associated with slow development of low levels of acquired protective immunity, parasite control relies on repeated use of dewormers (anthelmintics). Benzimidazoles (BZ) are currently the only anthelmintic registered in the EU for use in controlling *A*. *galli* and there is an obvious risk of overuse of one drug class, selecting for resistance. Thus we developed a reference transcriptome of *A*. *galli* to investigate the response in gene expression before and after exposure to the BZ drug flubendazole (FLBZ). Transcriptional variations between treated and untreated *A*. *galli* showed that transcripts annotated as mitochondrial glutamate dehydrogenase and cytochrome P450 were significantly down-regulated in treated worms, whereas transcripts homologous to heat shock proteins (HSP), catalase, phosphofructokinase, and a multidrug resistance P-glycoprotein (PGP1) were significantly up-regulated in treated worms. Investigation of candidate transcripts responsible for anthelmintic resistance in livestock nematodes led to identification of several tubulins, including six new isoforms of beta-tubulin, and several ligand-gated ionotropic receptors and ABC-transporters. We discovered several transcripts associated with drug binding and processing genes, but further characterisation using a larger set of worms exposed to BZs in functional assays is required to determine how these are involved in drug binding and metabolism.

## Introduction

Nematodes (roundworms) are elongated, cylindrical and bilaterally symmetrical organisms surrounded by a non-cellular cuticle over a layer of muscle cells covering a pseudocoel filled with an intestine and genitalia [1]. Genome studies have demonstrated extensive diversity within this phylum, including both parasitic and free-living genera, due to adaptation to diverse living conditions worldwide [2]. Parasitic genera can vary in length from millimetres up to half a meter and most species have both external and internal larval stages, as well as adults of both sexes, which are supported by a nervous system running along the length of the body [1].

However, even the gastro-intestinal nematodes of veterinary importance are not a taxonomically unified group. For example, nematode species causing health and welfare problems in livestock ruminants and equines are primarily found in clade V (Strongylida), whereas those of monogastric animals (e.g. birds, cats, dogs, humans and pigs) and one genus (*Parascaris*) of horses are mainly in clade III among the Ascaridida [3]. The genome of nematodes ranges in size from 50 to 250 Mb [4], but the variation across this phylum is probably even larger because genome size has so far only been estimated for a limited number of species. For example, genome size was recently shown to be 312 Mb in barber’s pole worm, *Haemonchus contortus* [5]. This is a highly pathogenic nematode of sheep and thus one of the best studied parasitic nematodes [6]. However, the genome is nearly seven times larger (~2100 Mb) in the ascarid *Parascaris equorum* of horses [7], illustrating a remarkable/significant variation in genome size between different species.

The genus *Ascaridia* belongs to the phylum nematoda and *A*. *galli* parasitises a wide range of domestic birds, i.e. chicken (*Gallus gallus domesticus*), and wild birds. It has a direct life cycle involving adult worms in the host’s small intestine and free-living stage (eggs) in the environment [1]. Parasite egg excretion in the faeces starts approximately 5–6 weeks after infection [8]. This is followed by the *in ovo* development of the unembryonated eggs to the infective third larval stage (L3) in the external environment. The life cycle is completed when a new host ingests infective parasite eggs [9]. Once the eggs reach the small intestine, L3 hatch within 24 h and undergo a histotrophic phase before they reappear as lumenal larvae that soon develop into adults. *Ascaridia galli* occurs worldwide, but is particularly prevalent in poultry kept in housing systems where the birds have access to faeces [10]. Thus *A*. *galli* is more abundant in aviary systems than in old-fashioned industrial production facilities with battery cages. Several studies have reported that the flock prevalence of this parasite has increased recently, due to the recent European Union-wide ban on conventional battery cages [10, 11].

Gastrointestinal nematode (GIN) parasitism is a major veterinary concern worldwide in grazing livestock and especially in small ruminants [12]. Since the development of immunity to nematodes is often slow and inefficient, parasite control is usually achieved by frequent use of anthelmintic drugs [13]. Thus, treatment with anthelmintics is a cornerstone in most livestock and human nematode control programmes. However, lack of efficient control of GIN is becoming a serious concern, particularly in small ruminant industries, due to the widespread and rapid development of anthelmintic resistance (AR), which in some countries is present for nearly all drug classes [14].

Although the genetic basis underlying AR is poorly understood for many substance classes, there is an advanced level of knowledge concerning resistance to benzimidazoles (BZ) in strongyle nematodes of grazing livestock. For example, for *H*. *contortus* it was shown in the 1990s that BZ resistance is linked to a substitution in position 200 in the drug target gene coding beta-tubulin isotype 1 [15]. This seems to be a common mechanism for BZ resistance in closely related strongyles of ruminants, such as *Teladorsagia circumcincta*, *Trichostrongylus colubriformis* and *Cooperia oncophora* [16–19]. However, there is now strong evidence that other single nucleotide polymorphisms (SNP) in additional codons in other beta-tubulin isotypes may also be involved in BZ resistance in strongyles [20–23].

While the mechanism for selection of BZ resistance is well known for strongylid nematodes, similar information concerning resistance is much more limited for *A*. *galli* and other ascarids of companion animals, horses and humans. In fact, and in contrast to the situation in strongyles, it seems as though BZ anthelmintics do not exert selection pressure on the beta-tubulin genes of isotype 1 and isotype 2 in the ascarid *P*. *equorum* [24]. This illustrates the difficulties in generalising about AR mechanisms between different taxonomic groups.

It is difficult to conduct research on the mechanisms involved in drug binding and metabolism until information on genes that may be involved becomes available. To date, only a few molecular studies on *A*. *galli* have been performed and have been limited to individual genes [24, 25]. Here, we present a *de novo* transcriptome assembly approach for an ascarid species using Illumina short-read sequence data. The aim was to investigate the gene expression levels in adult females of the poultry roundworm *A*. *galli* before and after exposure to FLBZ. Insights into these processes are of fundamental importance and can serve as a basis for identification of molecular targets in the rational design of sensitive genetic markers that can be applied for screening for AR genes in *A*. *galli*. Since there is currently an obvious risk of selection of AR in this parasite, we opted to explore genes that may be involved by using a combination of a candidate and non-candidate driven approach.

## Material and methods

### Ethical statement

The birds (Hy-Line breed) in this study originated from a commercial farm with laying hens with a history of *A*. *galli* infection. The flock contained approximately 7000 hens and were housed on concrete indoors. The barn was equipped with NATURA-Nova aviaries (big Dutchman) and fresh wood shavings were added as litter material before placement were fed a commercial layer ration (Fenix Topp pk bk, Lantmännen Lantbruk) and water was provided *ad libitum* during the flock cycle. Only soiled litter material was replaced during production. Handling of the birds and euthanasia were approved by the Swedish Ethical Committee for Scientific Experiments (C24/10). Birds were euthanized by stunning and cervical dislocation.

### Parasite material

Adult females of *A*. *galli* were collected from the small intestine of naturally infected laying hens before and on day three during a seven-day treatment period with FLBZ (Verminator^®^, Elanco Animal Health/Eli Lilly Danmark A/S) at 1.43 mg/kg body weight and day in accordance with the manufacturer’s recommendation. The hens were euthanized and live worms at both time points were collected from the small intestine as described elsewhere [8]. Three independent biological replicates from different hens were collected on both occasions. The worms were washed extensively in phosphate-buffered saline (PBS) (22°C) and then immediately submerged in RNA-later (Sigma-Aldricht), before being individually transferred into cryo-vials (Sigma-Aldricht) and frozen at -80°C.

### Sample preparation and sequencing

Total RNA was extracted and purified from the anterior end of *A*. *galli* female worms using the Qiagen RNeasy Kit according to the manufacturer’s protocol. A DNase treatment step using the RNase-Free DNase Set (Qiagen) was included to ensure RNA purity. After quality and quantity checks, SciLifeLab Uppsala performed library preparation and sequencing. The libraries were prepared from 1 μg total RNA using the TruSeq stranded mRNA sample preparation kit (Cat RS-122-2101/2102, Illumina Inc.) including polyA selection and clustered onto a HiSeq v4 flow cell using the Illumina cBot. The transcriptome sequencing was performed on the Illumina HiSeq2500 platform, using a 125 base pairs (bp) sequencing run generating 173 million paired-end reads. The sequence reads used in this study have been submitted to the ArrayExpress [26] public database at EBI under accession number E-MTAB-5705.

### *De novo* transcriptome assembly

The quality of the raw reads was assessed using the FastQC software v0.11.5 (http://www.bioinformatics.babraham.ac.uk/projects/fastqc/). The adapter was trimmed for each sample individually by applying Trimmomatic v0.32 [27], and only reads longer than 36 base pairs were retained for further analysis. The trimmed reads across all six samples were combined into a single dataset and *de novo* assembled with Trinity v2.4.0 [28]. Trinity was run both, in strand-specific mode (using the “—SS_lib_type RF” option), and in non-strand-specific mode. The result obtained by the non-strand-specific mode was used for subsequent analysis. The assembly quality and completeness was assessed by computing the E90N50 value and TransRate (v1.03) score [29], by examining full-length coverage against the Swissprot database and against all known *C*. *elegans* proteins, and by determining the strand specificity. In addition, the conserved ortholog content was identified using the nematod Benchmark Universal SingleCopy Orthologs (BUSCOs, v2.0.1) [30].

### Transcript abundance estimation and gene-expression analysis

Due to the lack of a fully assembled *A*. *galli* reference genome, the results of the *de novo* transcriptome assembly were used as reference to perform a sample-specific expression analysis. This was done using individual tools embedded in the Trinity differential expression module [31]. The original reads were mapped back against the reference transcriptome on a per sample basis using Bowtie2 [32], followed by calculation of abundance estimates using RSEM [33]. The read counts matrix obtained was used together with the Bioconductor edgeR package [34] to identify differential expression levels between treated and untreated samples. The data were normalised using the trimmed mean of M-values (TMM) normalisation method, which was corrected for library size and reduced RNA compositional effect [35]. Only genes represented with an adjusted P-value (FDR) lower than 0.001 and at least a four-fold change were considered as significantly differentially expressed in the pairwise comparison of the samples before and after FLBZ exposure.

### Functional annotation of the transcriptome

The Trinotate suite (http://trinotate.github.io/) was used to retrieve the functional annotation of the *A*. *galli* transcriptome. First, the Trinity transcripts were scanned for coding regions by TransDecoder (http://transdecoder.sf.net) [31], and all six-frame translations were filtered for a minimum length of 100 amino acids for open reading frames. The peptides identified were then further characterised based on sequence similarity against the non-redundant Uniref90 and SwissProt databases, using Blast (e-value < 1e-03). Based on the similarity search results, the transcripts were annotated with GO terms, Kegg pathways [36] and EggNOG (evolutionary genealogy of genes: non-supervised orthologous groups) [37]. Second, several functional annotation methods were applied to assess the functional data of *A*. *galli*. The HMMER approach and the Pfam database [38, 39] were used to identify protein domains, SignalP v4.1 [40] was used to predict the presence of signal peptides, the TMHMM server v2.0 [41] was used to predict transmembrane regions and RNAMMER v1.2 [42] was applied to identify rRNA transcripts. Third, the individual results were integrated into a SQLite database and combined to create an annotation report. Several filtering steps were then applied to the annotated transcripts, in order to identify true canonical genes. Only transcripts with the best open reading frame (ORF) and, in the case of splice variants, the longest transcript per gene were taken into consideration. The best ORF per transcript was obtained by favouring those with an assigned Pfam domain. In the absence of predicted Pfam domains or if all had an assigned Pfam domain, the longest available ORF for a transcript was selected. Transcripts without an ORF and without any functional characterisation were filtered out.

To facilitate identification of relevant functional genes, the remaining transcripts were divided into ‘low’ and ‘high’ confidence transcripts. Low confidence transcripts (LC) are supported either by an ORF alone, or by a Blast match against reference databases such as SwissProt and Uniref90, while high confidence transcripts (HC) are supported by both. Furthermore, both groups were classified by a keyword search of their associated Pfam and/or Blast description lines, into four categories: transposons (e.g. reverse transcriptase, transposase), potential transposons (e.g. zinc finger), genes with unknown function, and genes with an assigned function.

### Identification of potential candidate genes associated with BZ

Based on sequence similarity to the SwissProt reference database (BLASTP), a search was made for transcript candidates with matches to various proteins such as alpha, beta and gamma tubulins (TBA, TBB, TBG), multidrug resistance-associated protein (MRP), permeability glycoproteins (PGP), cytochromes P450 (CYP) and ligand-gated ion channels (LICs). Only matches with at least 50% sequence similarity were considered and extracted.

### Phylogenetic analysis of beta-tubulins

To determine which beta-tubulin isoforms were identified and how they are related to other known beta-tubulin sequences from closely related parasite species, a phylogenetic analysis was conducted. The identified protein sequences of the beta-tubulin candidates were aligned against the beta-tubulin proteins of *C*. *elegans* (Ce, 5 homologs), *Ascaris suum* (Asu, 3 homologs), *A*. *galli* (Asg, 1 homolog), *Haemonchus contortus* (Hco, 4 homologs), *Parascaris equorum* (Peq, 3 homologs) and *Trichinella spiralis* (Tsp, 1 homolog). Multiple sequence alignment was performed by the MAFFT L-INS-I iterative refinement method with local pairwise alignment information [43]. The alignment obtained was used to reconstruct the phylogeny selecting an evolutionary model based on the Bayesian information criterion (BIC) using the software prottest3 (v.3.4.2; https://github.com/ddarriba/prottest3), [44] and maximum likelihood inference using PhyML (v.3) [45]. The best fit model according to the BIC criterion was the JTT model [46], assuming a proportion of invariable sites (I) and rate variation modelled according to a gamma distribution (G). The branch support is given as bootstrap sampling proportions. The tree was rooted using the *Trichinella spiralis* beta-tubulin isoform as outgroup and visualised using the software FigTree (http://tree.bio.ed.ac.uk/software/figtree/).

## Results

### Sample sequencing and *de novo* assembly

Using the Illumina HiSeq2500 platform, a total of 172,984,619 paired-end raw reads with a length of 125 bp were generated from the three female *A*. *galli* worms before and after FLBZ treatment. After identification and removal of adapters and low-quality sequences from the reads, a total of 172,923,657 cleaned reads were retained, with a read length ranging from 36 bp to 125 bp. The reads were first assembled running Trinity in strand-specific mode. The strand-specificity examination of the resulted assembly showed that the strand-specific sequencing did not work as expected. Thus, the assembly was redone using Trinity in the non-strand-specific mode. From the roughly 173 million raw read pairs, a reference transcriptome assembly was generated with 301,681 contigs totalling 327 million base pairs with an average contig length of 2,337 bp and an E90N50 statistic of 2,342 nucleotides (S1 Fig). In total 6,291 out of 301,681 transcripts were included in this top-90% subset, indicating that a large amount of the assembled transcripts were expressed at rather low levels. The longest contig obtained covered 14.6 kb (Table 1).

**Table 1:** *De novo* assembly and annotations metrics for the transcriptome of *Ascaridia galli*.

	*Ascaridia galli*
Assembled transcripts	301,681
Assembled Trinity”genes”	162,400
Assembled bases	327,057,823
Contig N50	2,337
Contig E90N50	2,342
Average length	1084.12
Longest contig	14,656
Shortest contig	201
GC%	42.04
Transcripts with ORF	119,701
Transcripts with annotation	30,959
Annotated transcripts with ORF	17,230
Pfam	8,786
Signal peptides	5
Transmembrane domains	3,901
Ribosomal RNA (RNAMMER)	10
EggNOG	9,786
KEGG	9,919
Gene ontology	8,877
SwissProt	9,502

The assembly completeness and accuracy has been assessed by calculating the BUSCO and TransRate scores, as well as by examining the amount of full-length reconstructed transcripts. The assembled *A*. *galli* transcripts were compared against the benchmarking universal single-copy orthologs (BUSCO) for nematode species revealing that 91.9% of the BUSCO groups have complete gene representation, while 4.8% are only partially recovered, and 3.3% are missing. TransRate measures how well the assembled transcripts are supported by the raw RNA-Seq reads, reporting scores ranging between 0 and 1. The TransRate score obtained for the present assembly is 0.015, while the reported optimal score (which might be achieved by removing bad scoring transcripts) is 0.039. This scores seems to be in accordance with other reported TransRate scores for de novo transcriptome assemblies [29] and suggest that it is unlikely to improve the quality of the present assembly without additional data. The sequence comparison of the assembled transcripts against the Swissprot database and the available *C*. *elegans* proteins showed that there are 3843 Swissprot and 5204 *C*. *elegans* proteins that are represented by nearly full-length transcripts having > 80% alignment coverage.

### Functional annotation of the *A*. *galli* transcriptome

Initially, 119,701 protein-coding Trinity isoforms were reported by Transdecoder (S1 File, S2 File), which were filtered to retrieve only one single best ORF per transcript, with potential genes with longer ORFs preferred. In total, 75.9% of the predicted ORFs (90,934 protein-coding genes) were kept for further analysis and classified according to their completeness: 67.5% with full ORF (complete), 21.6% with missing start codon (5’ partial), 5.8% with missing stop codon (3’ partial) and 5.1% with no start or stop codon detected (internal).

A total of 30,959 (19.1%) transcripts were annotated, of which 17,230 had an open reading frame. The remaining 131,441 (80.9%) transcripts had neither a functional annotation nor an ORF (S1 Table). Approximately 51% (8,786) of the transcripts with an ORF were associated with a protein domain, 3,901 contained a transmembrane helix, while ten were predicted as ribosomal RNA genes (Table 1). The transcripts lacking a functional annotation assignment and ORF could be explained by their location in untranslated regions, their inability to encode a protein (non-coding RNAs) or the lack of proper annotation and/or a protein domain. Roughly 85% of the transcripts having an ORF lack a functional annotation. These transcripts are good candidates either for *A*. *galli* specific genes, which might be correlated with key life history functions [2], or nematode specific pseudogenes.

### Classification of annotated transcripts

**Fig 1:**
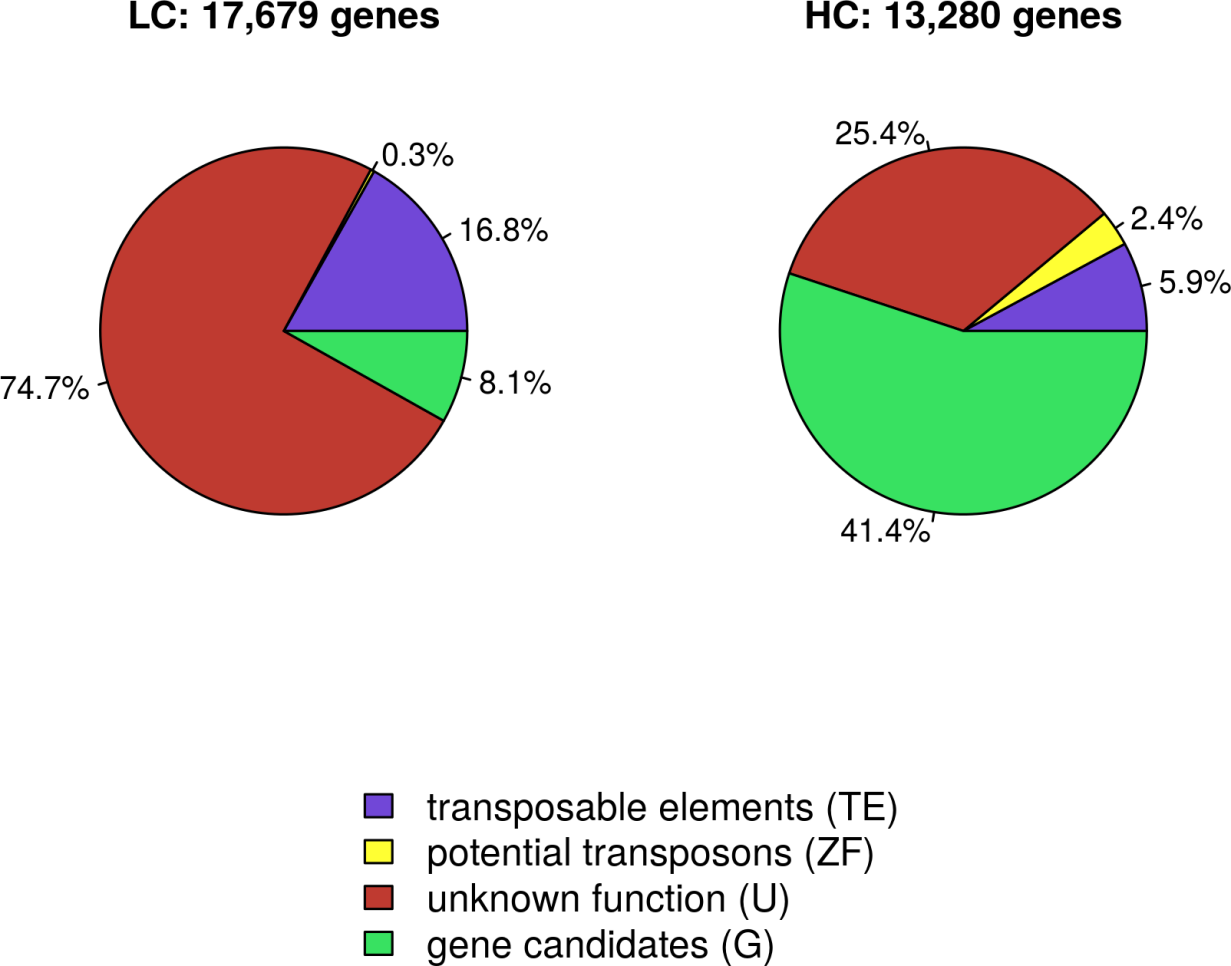
Classification of annotated transcripts. The 30,959 annotated transcripts were grouped in two classes, LC = low confidence (17,679 transcripts) or HC = high confidence (13,280 transcripts), based on the quality of their annotation. Using a keyword search of their Blast and Pfam description line, the transcripts of each class were further assigned to the following four categories: transposable elements (TE, e.g. transposon, retrotransposon), potential transposons (e.g. zinc finger, ZF), genes with unknown function (U) and genes with an assigned function (G).

About half of the 30,959 transcripts (57%) were classified as ‘low confidence’ (LC), the other half as ‘high confidence’ (HC). A keyword search of their Pfam and Blast description line revealed that 5.9% (HC) and 16.8% (LC) of the transcripts were categorised as transposable elements. Potential transposons (e.g. zinc finger) marked 2.4% (HC) and 0.3% (LC) of the transcripts, while 25.4% (HC) and 74.7% (LC) were characterised as genes with unknown function. The remaining transcripts were categorized as potential genes (Fig 1).

### Identification of candidate transcripts associated with drug binding and processing

Based on sequence similarity, several transcripts associated with drug binding and processing genes were identified, among which were six candidates of beta-tubulins, four candidates of ligand-gated ion channels, two candidates of cytochromes P450, and four candidates of membrane-associated transport permeability glycoproteins (PGP) (Table 2). All of the candidates identified displayed at least 50% sequence similarity to the corresponding reference proteins and thereby provided a good basis for further investigations. Furthermore, both cytochromes P450 candidates and one of the PGP candidates were significantly down- and up-regulated, respectively.

**Table 2:** Number of identified candidate transcripts associated with drug binding and processing.

Transcript	No. of candidates
Alpha tubulin	8
Beta tubulin	6
Gamma tubulin	1
Multidrug resistance P-glycoprotein (PGP)	4
Cytochrome P450 (CYP)	2
Multidrug resistance-associated protein (MRP)	2
Ligand-gated ion channel (LICs)	4

### Analysis of differentially expressed transcripts

**Fig 2:**
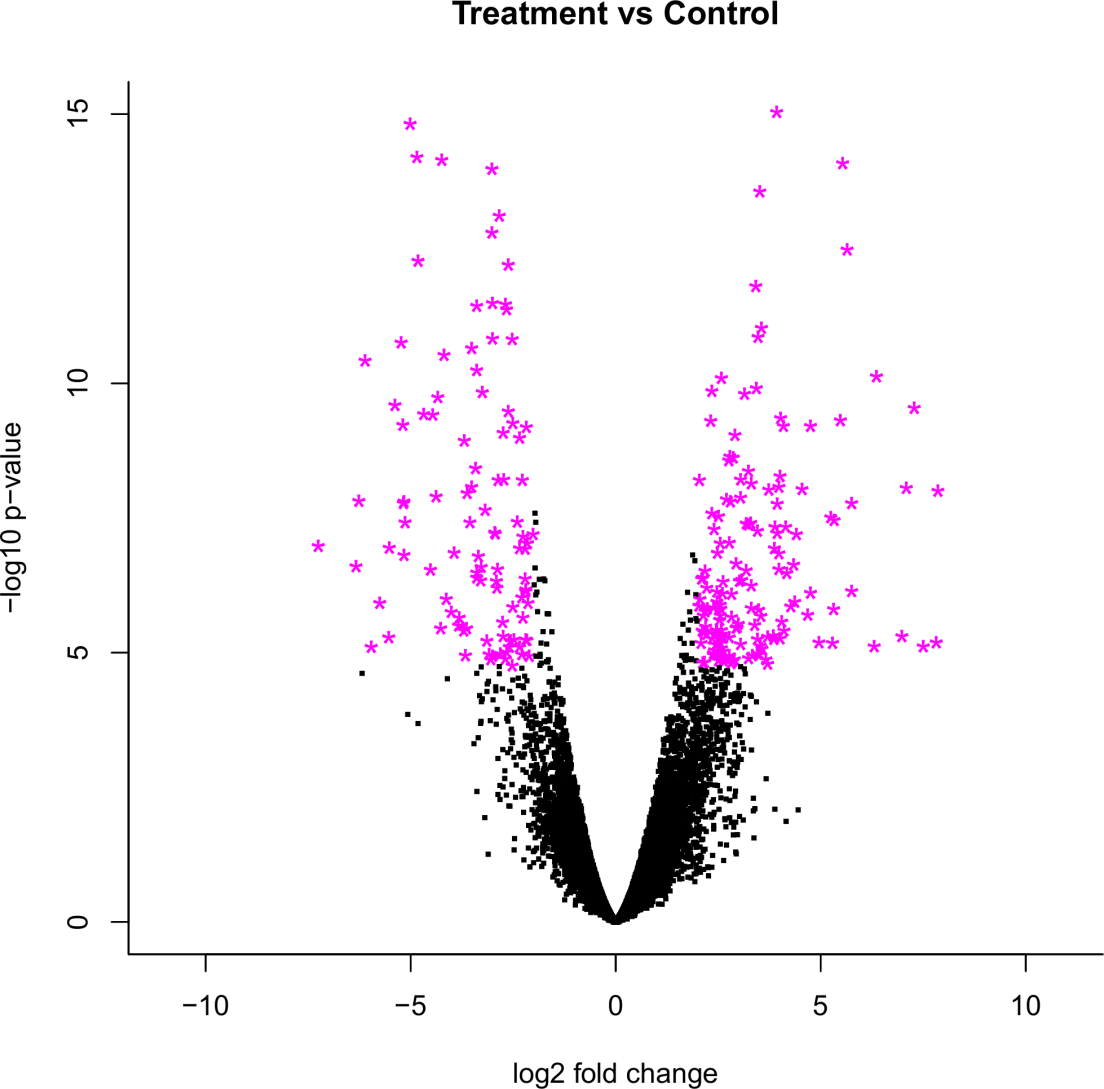
Volcano plot of all differentially expressed transcripts. Purple dots denote significantly differentially expressed transcripts with an adjusted p-value lower than 0.001 and at least 4-fold change.

Comparisons of gene expression levels in *A*. *galli* exposed to FLBZ were carried out to identify transcript candidates that responded to FLBZ treatment. In total, 263 significantly differentially expressed (DE) transcripts were detected using stringent filtering criteria: an adjusted p-value (FDR) lower than 0.001 and a log fold change above 2 (or below -2) (Fig 2). Of these, 110 (41.8%) transcripts were up-regulated in the FLBZ-treated samples, while 153 (58.2%) were down-regulated. Based on the quality of the functional annotation 67% out of 263 differentially expressed transcripts were classified as high confidence DE transcripts, while 33% of the transcripts lacked any annotation (Table 3). Moreover, the high confidence DE transcripts were grouped into genes with known function (57.9%), genes with unknown function (34.6%), transposable elements (6.3%), and potential transposons (1.2%) (S2 Table). The remaining 33% significantly differentially expressed transcripts have neither a functional annotation nor a predicted open reading frame, suggesting that these ‘orphan’ transcripts might be transcribed (possibly nematod specific) non-coding RNAs.

**Table 3:** Classification of differentially expressed genes.

	Down-regulated	Up-regulated
No. of regulated genes	153	110
No. of genes without annotation	48	39
No. of high confidence (HC) genes:	105	71
Genes with known function	61	41
Genes with unknown function	37	24
Transposable elements	7	4
Potential transposons	0	2
No of low confidence (LC) genes:	0	0

Classification of significantly up- and down-regulated differentially expressed genes in the FLBZ-treated *Ascaridia galli* samples.

### Phylogenetic analysis of the *A*. *galli* ß-tubulin sequences

**Fig 3:**
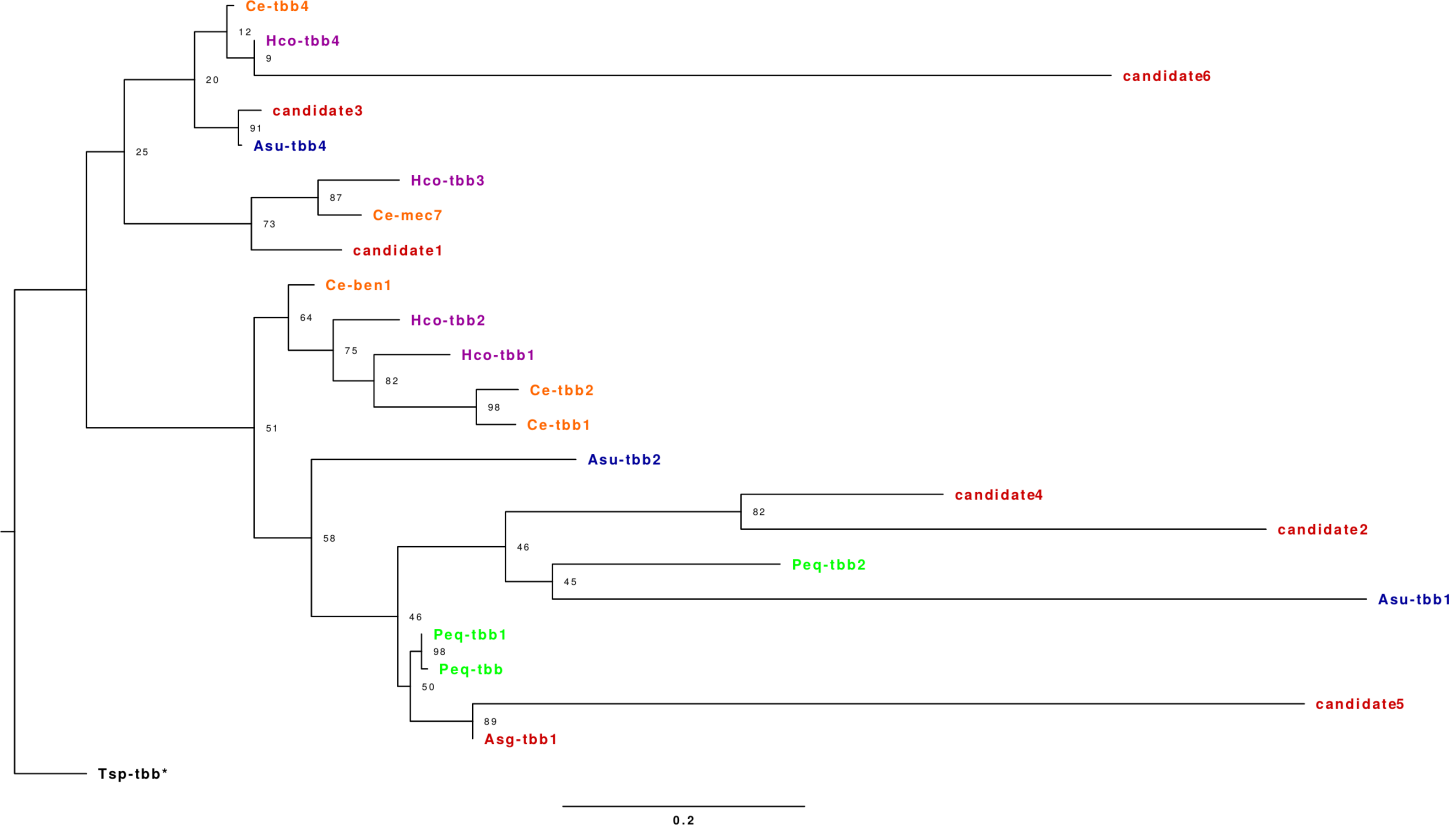
Phylogenetic tree of beta-tubulin genes. The phylogram was obtained by maximum likelihood analysis of beta-tubulin genes across six species: *Caenorhabditis elegans* (Ce), *Haemonchus contortus* (Hco), *Parascaris equorum* (Peq), *Ascaris suum* (Asu), *Trichinella spiralis* (Tsp) and *Ascaridia galli* (Asg). The tree was constructed by model selection based on the BIC criterion using the software prottest3, and maximum likelihood inference using PhyML. The branch support is given as bootstrap sampling proportion (the closer to 100, the more confident). The tree was rooted using the species *T*. *spiralis* as outgroup. Beta-tubulin isoforms belonging to the same species are indicated by colour: *H*. *contortus* is shown in purple, *A*. *suum* in blue, *C*. *elegans* in orange, *P*. *equorum* in green, *T*. *spiralis* in dark green and *A*. *galli* candidates in dark red.

The phylogenetic analysis included reference beta-tubulin sequences from six different nematode species, namely: *C*. *elegans–*isotypes 1, 2, 4, ben1 and mec7, *A*. *galli–*isotype 1, *A*. *suum–*isotypes 1 and 4, *H*. *contortus–*isotypes 1 to 4, *P*. *equorum–*isotypes 1 and 2 and *T*. *spiralis*–isotype 1, as well as six beta-tubulin candidate transcripts identified in this study (S3 Table). The aligned protein sequences allowed reconstruction of a maximum likelihood phylogenetic tree and assignment of the six *A*. *galli* beta-tubulin candidates to isotypes. Four out of six candidate transcripts formed clades with the isotype 1 of *A*. *galli* and *P*. *equorum* (candidate 5), isotype 3 of *H*. *contortus* and *C*. *elegans mec7* (candidate 1) and isotype 4 of *C*. *elegans*, *H*. *contortus* and *A*. *suum* (candidate 3 and 6). The remaining two candidates (candidates 2 and 4) formed a clade with the isotype 1 of *A*. *suum* and isotype 2 of *P*. *equorum*, but showed a low confidence value, impeding distinct classification (Fig 3). Furthermore, within the phylogram the beta-tubulin isotypes 1 and 2 of *C*. *elegans* and *H*. *contortus* grouped in one clade, which could be an indicator of mis-labelling of these reference genes.

## Discussion

Baseline information about gene expression is of fundamental importance for ongoing research on identification of mechanisms involved in the uptake and metabolism of anthelmintic drugs in target nematodes. Compared with other nematodes of veterinary concern, few genomic resources and molecular markers are available for the poultry roundworm *A*. *galli*. We therefore investigated differences in gene expression levels in worms collected before and after FLBZ exposure, in order to identify genes that may be of particular concern for the mode of action of this group of anthelmintics and how these may be involved in metabolic pathways and eventually selection of resistance.

We used *de novo* transcriptome sequencing and assembly here to investigate the genetic response in *A*. *galli* before and after FLBZ exposure. We believe that this is the first attempt at using Illumina paired-end sequencing technology to reconstruct and analyse the *A*. *galli* transcriptome. The transcriptome data proved to be valuable material, providing insights into the genes involved in the response mechanisms of *A*. *galli* to FLBZ exposure. They provided an excellent source for mining and development of gene-associated markers. Roughly 173 million high quality paired-end reads were used for *de novo* assembly in non-strand-specific mode, generating 301,681transcriptome contigs covering 162,400genes. The non-strand-specific mode was selected after examining the strand-specificity, which showed that the strand-specific sequencing was not successful. The applied assembly quality metrics (e.x. BUSCO, TransRate) on the non-strand-specific assembled transcriptome revealed that despite its fragmentation, it covers over 91% of the nematod specific BUSCO groups and roughly 19% of the *C*. *elegans* (WBcel235) proteins to over 80% of their full length.

About 19.1% (30,959) of the transcripts mentioned above could be functionally characterised and annotated, among which 17,230 (55.6%) exhibited an open reading frame (ORF). The large proportion of uncharacterised transcripts might be attributed to non-coding RNAs, transcribed transposable elements, nematode specific genes, or pseudogenes. The lack of proper annotation for most nematode worms might also play a role. Nonetheless, this *A*. *galli* specific transcripts might play an important role in drug responses and mechanisms of drug tolerance, providing a valuable resource in understanding how *A*. *galli* responds to FBLZ treatment and should be further explored.

Based on the quality of their annotation, these transcripts were further filtered and classified, reducing the number of transcripts to 13,280 high confidence transcripts. Of these, 176 (1.3%) were significantly differentially expressed in response to drug exposure.

Interestingly, among the transcripts that were significantly differentially expressed using the strict criteria applied here, a transcript annotated as mitochondrial glutamate dehydrogenase (GDH) was found to be significantly down-regulated following FLBZ exposure. GDH is located in the mitochondria and is an important branch-point enzyme which has a key role in nitrogen and glutamate (Glu) metabolism and energy homeostasis in other nematodes [47]. A prominent role of mitochondria is to provide energy currency for the cell and to cellular event reactions involved in ATP production [48]. This indicates that drug-exposed worms were severely affected by FLBZ and showed signs of dying, which was confirmed some days later [8]. These observations are basically in line with Hanser et al [49], who showed extensive drug-derived cellular destruction including disruption of mitochondria in the hypodermis, intestine and muscle cells in two closely related species of roundworms after exposure to FLBZ. Similarly, both, heat shock proteins (HSP) and a permeability glycoprotein (PGP) were significantly up-regulated as a result of drug exposure. The HSP are a subset of a larger group of proteins produced in most organisms in response to exposure to a range of stressful conditions [50]. All of the above-described genes are somehow involved in cell death and are thus of importance for worm survival. It would be worthwhile investigating how their transcription levels could be utilised as endpoints in *in vitro* bioassays measuring the susceptibility of eggs or adult worms in response to gradually increasing concentrations of FLBZ.

While the precise mode of action of BZs, including FLBZ, in *A*. *galli* is not yet fully understood, it is anticipated that these substances act by inhibiting tubulin polymerisation. Therefore, we further analysed the beta-tubulins and several other genes that may be involved in the various pathways of BZ anthelmintic action. In previous research, only one isotype of beta-tubulin has been described in *A*. *galli* [24]. However, we identified six novel *A*. *galli* beta-tubulin paralogs (so-called beta-tubulin candidates), of which candidate 5 in this study is most similar in its sequence to the previously identified *Aga-tbb-iso-1* [24]. Overall, we found that the degree of beta-tubulin gene diversity in *A*. *galli* is in agreement with that in several other well-studied nematode species [51, 52]. According to the present study, isoforms 1 and 2 of *C*. *elegans* and *H*. *contortus* are obviously closer to each other than the corresponding isotypes of other ascarids such as *A*. *galli*, *A*. *suum* and *P*. *equorum*. This is consistent with previous suggestions and provides further evidence that the mode of action of anthelmintics in the model nematode *C*. *elegans* may have more in common with strongylid parasites such as *H*. *contortus*, *Cooperia oncophora* and *Ostertagia ostertagia* than more distant relatives found among ascarids [24]. However, no differences were found when we compared the gene expression levels of different beta-tubulin isotypes from treated and untreated worms. This has been observed previously in the filaroid nematode *Brugia malayi* after FLBZ exposure [53]. Although it has been shown that certain mutations are implicated in BZ resistance in strongylid nematodes [21, 22, 52], further investigation is required to confirm whether a similar mechanism is involved in the drug binding and selection for AR in ascarids, including *A*. *galli*.

Besides the direct action of the drug due to binding of FLBZ to beta-tubulin, it is believed that the depolymerisation of microtubules may have other additional effects, including inhibition of cellular transport and energy metabolism. Here we found that both phosphofructokinase and catalase were significantly up-regulated in worms exposed to FLBZ. Phosphofructokinase is a universal enzyme that serves as a key regulatory step in the glycolysis pathway and is of fundamental importance for carbohydrate catabolism. The enzyme-catalysed transfer of a phosphoryl group from ATP is an important reaction in a wide variety of biological processes, for example in *A*. *suum* [54]. Catalase is another common enzyme found in nearly all living organisms exposed to oxygen, including *C*. *elegans*, where it is responsible for the quenching of hydrogen peroxide to water and oxygen and is thereby involved in protection of the cell from oxidative damage by reactive oxygen species [55]. The increased expression of these gene products in response to drug exposure suggests that central functions in the glycolysis pathway of *A*. *galli* are affected. Although we only identified one gene in this pathway, which encodes for phosphofructokinase, our data may facilitate future study of related genes.

In addition, several cell-bound cytochromes P450 (CYP) (significantly down-regulated), membrane-bound P-glycoproteins (PGP) efflux pumps (significantly up-regulated) and multidrug resistance-associated proteins (MRP) were identified. Apart from CYP, these genes are members of the superfamily of ATP-binding cassette (ABC) transporters, which are known to transport various xenobiotic toxic molecules across extra- and intra-cellular membranes. These can thereby actively remove drugs, thus preventing their accumulation at the cellular level. Although there are few functional studies in nematodes, ABC transporters have previously been identified in nematode genomes, where they are known to confer resistance [56]. Thus some of the transporters identified here can be used as putative genetic markers to monitor drug resistance.

The present study showed that the *A*. *galli* transcriptome assembly provides a major genomic resource in understanding the response of chicken roundworms to FLBZ exposure. The identified down-regulated (mitochondrial glutamate dehydrogenase, cytochromes P450) and up-regulated (heat shock proteins, catalase, phosphofructokinase, and permeability glycoproteins) transcripts, as well as the six new isotypes of beta-tubulins and several ABC transporters, can be used as putative/possible tools in genetic marker development. They can provide the basis for further research identifying drug metabolism in nematodes and monitoring drug resistance. In conclusion, this study substantially increases understanding of how *A*. *galli* responds to BZ anthelmintics.

## Supporting information

S1 Fig Assembly quality metrics. The plot visualizes in the left panel (A) the distribution of the orientation ratio for each contig in the transcriptome assembled in non-strand-specific mode. The right panel (B) visualizes the N50 statistic for the top most highly expressed transcripts that represent x% of the total normalized expression data. The maximum value in our data set is found near E95 with an N50 of 2,729 and 22,496 transcripts.
(PDF)

S1 Table Transcriptome annotation with Trinotate for all transcripts. (XLSX)

S2 Table Annotation of differentially expressed transcripts classified with high confidence (HC) (XLSX)

S3 Table Beta-tubulin isoform abbreviations. (XLSX)

S1 File The gff3 file of the predicted ORFs by Transdecoder. (ZIP)

S2 File The protein sequences of the predicted ORFs by Transdecoder. (ZIP)
