# Supplementary figures and images for: RNA-Seq de novo assembly and differential transcriptome analysis of the nematode Ascaridia galli in relation to in vivo exposure to flubendazole
Source: PLoS One. 2017 Nov 3;12(11):e0185182. doi: 10.1371/journal.pone.0185182 (PMC5669496; doi:10.1371/journal.pone.0185182)

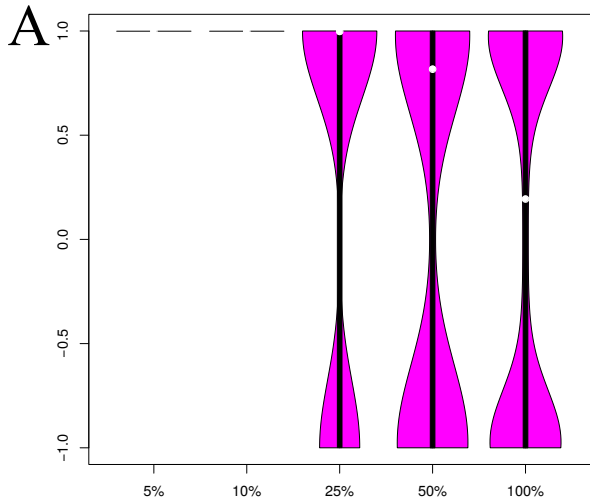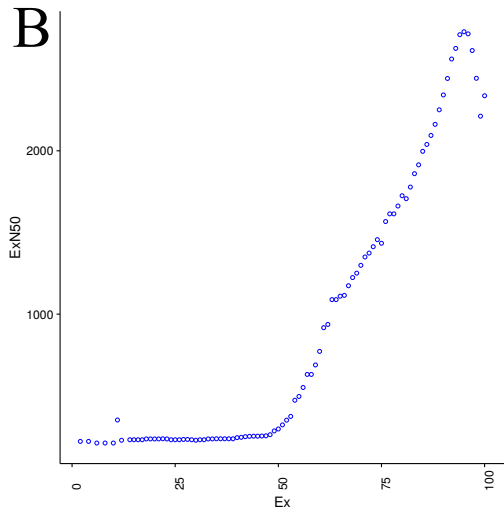

Supplement: S1 Fig — The plot visualizes in the left panel (A) the distribution of the orientation ratio for each contig in the transcriptome assembled in non-strand-specific mode. The right panel (B) visualizes the N50 statistic for the top most highly expressed transcripts that represent x% of the total normalized expression data. The maximum value in our data set is found near E95 with an N50 of 2,729 and 22,496 transcripts. (PDF) [file pone.0185182.s001.pdf]
